# Microbial Community Diversity Within Sediments from Two Geographically Separated Hadal Trenches

**DOI:** 10.3389/fmicb.2019.00347

**Published:** 2019-03-15

**Authors:** Logan M. Peoples, Eleanna Grammatopoulou, Michelle Pombrol, Xiaoxiong Xu, Oladayo Osuntokun, Jessica Blanton, Eric E. Allen, Clifton C. Nunnally, Jeffrey C. Drazen, Daniel J. Mayor, Douglas H. Bartlett

**Affiliations:** ^1^ Marine Biology Research Division, Scripps Institution of Oceanography, University of California San Diego, La Jolla, CA, United States; ^2^ Oceanlab, The Institute of Biological and Environmental Sciences, King’s College, The University of Aberdeen, Aberdeen, United Kingdom; ^3^ Louisiana Universities Marine Consortium (LUMCON), Chauvin, LA, United States; ^4^ Department of Oceanography, University of Hawai’i at Ma-noa, Honolulu, HI, United States; ^5^ National Oceanography Centre, University of Southampton Waterfront Campus European Way, Southampton, United Kingdom

**Keywords:** hadal, trench, sediment, pressure, piezophile

## Abstract

Hadal ocean sediments, found at sites deeper than 6,000 m water depth, are thought to contain microbial communities distinct from those at shallower depths due to high hydrostatic pressures and higher abundances of organic matter. These communities may also differ from one other as a result of geographical isolation. Here we compare microbial community composition in surficial sediments of two hadal environments—the Mariana and Kermadec trenches—to evaluate microbial biogeography at hadal depths. Sediment microbial consortia were distinct between trenches, with higher relative sequence abundances of taxa previously correlated with organic matter degradation present in the Kermadec Trench. In contrast, the Mariana Trench, and deeper sediments in both trenches, were enriched in taxa predicted to break down recalcitrant material and contained other uncharacterized lineages. At the 97% similarity level, sequence-abundant taxa were not trench-specific and were related to those found in other hadal and abyssal habitats, indicating potential connectivity between geographically isolated sediments. Despite the diversity of microorganisms identified using culture-independent techniques, most isolates obtained under *in situ* pressures were related to previously identified piezophiles. Members related to these same taxa also became dominant community members when native sediments were incubated under static, long-term, unamended high-pressure conditions. Our results support the hypothesis that there is connectivity between sediment microbial populations inhabiting the Mariana and Kermadec trenches while showing that both whole communities and specific microbial lineages vary between trench of collection and sediment horizon depth. This *in situ* biodiversity is largely missed when incubating samples within pressure vessels and highlights the need for revised protocols for high-pressure incubations.

## Introduction

Ocean sediments make up one of the largest biomes on earth, harboring an estimated 3 × 10^29^ total microbial cells distributed in 3 × 10^8^ km^3^ of sediment with 8 × 10^7^ km^3^ of pore water (Kallmeyer et al., 2012; Amend and LaRowe, 2016). Deep-sea sediment microbial community composition is influenced by organic matter abundance and content (D’Hondt et al., 2009; Bienhold et al., 2012, 2016; Kallmeyer et al., 2012; Jacob et al., 2013), sediment horizon depth (Walsh et al., 2016a,b), water column depth (Jacob et al., 2013), and geographical location (Hamdan et al., 2013; Bienhold et al., 2016). Sediment communities are distinct from those in the water column despite the deposition of sinking taxa from above (Zinger et al., 2011; Hamdan et al., 2013). While surficial sediments include high abundances of *Gammaproteobacteria*, *Deltaproteobacteria*, *Alphaproteobacteria*, and *Actinobacteria* (Zinger et al., 2011; Ruff et al., 2015), deeper subsurface layers are dominated by the *Chloroflexi* and *Atribacteria* (OP9/JS1; Bienhold et al., 2016; Walsh et al., 2016b). These deeper communities may consist of taxa adapted to deep subsurface conditions (Inagaki et al., 2015) or which are found at shallower sediment horizon depths and survive after burial (Starnawski et al., 2017).

Sediment microbial communities at hadal depths remain largely unexplored. These sites are deeper than 6,000 m water depth and are typically affiliated with trenches, steep-walled depressions formed through the subduction of one tectonic plate below another. Sediment oxygen concentrations can drop from 200 μmol l^−1^ to undetectable levels over the top 10 cm in trenches, indicating high rates of oxygen consumption (Glud et al., 2013; Wenzhöfer et al., 2016). Topographical funneling of organic matter may sustain this activity (Danovaro et al., 2003; Glud et al., 2013; Wenzhöfer et al., 2016) as increases in organic material with water depth have been observed in the Mariana Trench (Luo et al., 2017) and modeled in the Kermadec Trench (Ichino et al., 2015). An alternative source of organic carbon within trenches may be geochemical inputs from below (Li et al., 1999; Fujiwara et al., 2001; Tarn et al., 2016). Until recently, microbial community analyses of hadal sediments have been limited to 16S rRNA gene sequence studies with small sample sizes (Kato et al., 1997; Li et al., 1999; Yanagibayashi et al., 1999; Nunoura et al., 2013; Yoshida et al., 2013; Luo et al., 2015) and cultivation attempts. Culture-independent analyses have identified taxa affiliated with the same lineages as those found within other shallower deep-ocean sediments and suggested the importance of nitrogen cycling within these communities (Nunoura et al., 2013, 2018; Yoshida et al., 2013; Luo et al., 2015; León-Zayas et al., 2017). High-pressure, culture-based analyses have predominantly found copiotrophic members of the *Gammaproteobacteria*, including *Shewanella*, *Colwellia*, *Moritella*, and *Psychromonas* (Kato et al., 1998; Nogi et al., 1998, 2002, 2004, 2007).

Two of the deepest locations in the ocean are the Mariana and Kermadec trenches. The Mariana Trench, located in the Northern Hemisphere near the Mariana Islands, extends to 10,984 m at its greatest depth (Gardner et al., 2014). The Kermadec Trench begins off the northeastern coast of New Zealand and reaches a maximum depth of 10,047 m (Angel, 1982). These trenches reside approximately 6,000 km apart within the Pacific Ocean. Deep-sea sediments can show high levels of microbial endemism and significant decay of community similarity over distance (Zinger et al., 2014; Bienhold et al., 2016). Endemism could be especially prevalent in hadal trenches, which are predicted to be rich in endemic taxa due to their extreme depths and geographical isolation (Beliaev, 1989). Furthermore, water mass inputs and annual rates of primary production vary between the two trenches, with primary productivity in the overlying waters of the Kermadec estimated at 87 g C m^−2^ yr^−1^ compared to 59 g C m^−2^ yr^−1^ in the waters above the Mariana Trench (Longhurst et al., 1995; Jamieson, 2015). Therefore, we hypothesize that geographical isolation and differences in organic matter input lead to distinct community compositions between the two trenches. In this study, we investigated the microbial communities within surficial sediment (0–10 cm) samples collected from 6- to 9-km water depths in the Kermadec Trench and 7- to 8-km depths in the Mariana Trench with both culture-independent high-throughput 16S rRNA gene sequencing and culture-dependent characterization.

## Materials and Methods

### Sample Collection

Microbial community composition was evaluated within 88 total samples from the Mariana and Kermadec trenches. This included 72 samples belonging to 14 intact, depth-fractioned cores, 3 from the Mariana Trench at depths of 6,844–7,942 m and 11 from the Kermadec Trench ranging from 6,011 to 9,177 m (Supplementary Table 1). Kermadec Trench samples were collected on the R/V *Thompson* from April to May 2014, and Mariana Trench samples were collected during a cruise on the R/V *Falkor* from November to December 2014. Bathymetry was obtained from NOAA (Amante and Eakins, 2009) and plotted using the R package marmap (Pante and Simon-Bouhet, 2013). Sediment in the Kermadec Trench was collected using push cores with the HROV *Nereus* (Fletcher et al., 2009). Samples in the Mariana Trench were recovered using untethered free-falling/ascending landers (Free Vehicle Coring Respirometer (FVCR) and Rock Grabber (Schmidt Ocean Institute, https://schmidtocean.org/technology/elevators-landers/)). Large-diameter (10 cm) megacores were inserted into the seabed by the FVCR using an oil-compensated motor at a steady and slow speed 2 h after landing (Nunally et al., in prep). Megacore tubes were visibly inspected using a task camera on the FVCR to assure sediment integrity was maintained during coring operations. The Rock Grabber lander collected sediments using a Van-Veen Grab. After shipboard recovery, samples were immediately moved to a 4°C cold room to minimize the effects of temperature stress.

### Sample Processing

Sediment samples were depth fractioned from 0–1, 1–2, 2–5, and 5–10 cm in the Kermadec Trench and down to 10 cm at one-centimeter increments in the Mariana Trench. For samples collected using the Rock Grabber, subsamples were obtained using a sterile syringe inserted up to 10-cm depth, after which subsamples from the top 2–3 cm were extruded into KAPAK bags (Komplete Packaging, Grand Prairie, TX) and homogenized. Samples for DNA extraction were then frozen at −80°C. To determine the effects of long-term pressurization on microbial communities, ~5-g samples were also incubated at *in situ* pressure in KAPAK bags without amendment for 1 (Mariana) or 1.5 (Kermadec) years. Rock Grabber and other samples not part of intact cores were not included in our analysis of the *in situ* community as these samples were likely disturbed and mixed with deeper sediment layers and overlying water during ascent. Therefore, they were only used for culturing and experiments under long-term high-hydrostatic pressure conditions.

### DNA Extraction and Itag 16S rRNA Gene High-Throughput Sequencing

Sediment (5-g wet-weight) samples from either frozen samples or long-term *in situ* high-hydrostatic pressure incubations were used for DNA extraction. DNA was extracted using a modified version of Lysis Protocol II described by Lever et al. (2015). 2.5 V of lysis solution (30 mM EDTA, 30 mM Tris–HCl, 800 mM guanidine hydrochloride, 0.5% Triton X-100, final pH 10) and 500 μmol pyrophosphate was added to each sample and the mixture briefly vortexed. Samples were then subjected to two 15-minute freeze–thaw cycles at −80°C, followed by incubation at 50°C with shaking at 150 rpm for 1 h. Samples were centrifuged and the supernatant was treated twice with 1V 24:1 chloroform:isoamyl alcohol. DNA was precipitated using 5 M NaCl and 70% ethanol for 2 h at room temperature and resuspended in nuclease-free water. Extracted DNA was cleaned again using a Quick-gDNA MiniPrep kit (Zymo Research, Irvine, CA). Extraction blanks, consisting of all reagents but no sediment material, were performed in concomitance with every extraction.

### 16S rRNA Gene Sequencing and Statistical Analysis

Sequencing and processing were conducted as previously described (Peoples et al., 2018a). Briefly, the V4-V5 16S rRNA gene region between 515f-926R was amplified (Parada et al., 2015) for 30 cycles and tagged with Illumina barcodes using a secondary PCR procedure. Samples were pooled at equimolar concentrations and sent for sequencing on an Illumina Miseq at the Institute for Genomic Medicine Genomics Center (University of California, San Diego, La Jolla, CA). Paired-end reads were assembled using FLASH (Magoc and Salzberg, 2011) and OTUs picked at 97% similarity using Uclust in QIIME 1.9.1 (Caporaso et al., 2010). Chimeras were identified and removed using VSEARCH (Rognes et al., 2016) and taxonomy assigned against the SILVA 123 release database (Quast et al., 2012). Sequences found within sequenced extraction blanks and which showed similarity to known contaminants were discarded. The exclusion of contaminants was done manually to avoid removing any autochthonous taxa that may have resulted from cross-contamination between samples and controls. Removed genera included *Acinetobacter*, *Stenotrophomonas*, *Methylobacterium*, *Achromobacter*, *Rhizobium*, *Tetragenococcus*, *Escherichia-Shigella*, *Ferribacterium*, *Massilia*, *Curvibacter*, *Ralstonia*, *Variovorax*, *Deinococcus*, *Anoxybacillus*, *Aeribacillus*, *Brevundimonas*, *Enterobacter*, *Streptococcus*, *Burkholderia*, *Staphylococcus*, *Sphingomonas*, *Sphingobium*, *Sphingobacterium*, *Bradyrhizobium*, *Paenibacillus*, and the family *Comamonadaceae*. Raw sequence data can be accessed at the Sequence Read Archive under the Biosample Accession numbers SAMN07732241-SAMN07732328.

Sequencing reads were processed with the R package phyloseq (McMurdie and Holmes, 2013). OTUs were removed if they did not have an abundance of at least three reads in at least four samples across the entire dataset. Samples were rarefied to even sampling depth to account for differing sequencing depths between samples. Alpha diversity was calculated using the package vegan (Oksanen et al., 2017) and comparisons between samples were performed using the beta-diversity metric Bray-Curtis in phyloseq. Permutational analysis of variance with adonis was used to determine if trench of collection, sediment horizon depth, and water column depth significantly explained community variation within and between trenches. DESeq2 (Love et al., 2014) was used to identify taxa that were differentially abundant between trench of collection, sediment horizon depth (0–5 or 5–10 cm), water column depth (Kermadec only; 6,000 and 7,000 m vs. 8,000 and 9,000 m), and high-pressure comparison (samples maintained under long-term high hydrostatic pressures and the same samples extracted immediately). DESeq2 analyses were performed on the un-rarefied dataset (McMurdie and Holmes, 2014) with low-abundance (at least <500 total reads per OTU or < 1,000 reads per class or phylum) reads removed. For phylogenetic analysis, representative sequences were aligned with the SINA Aligner (Pruesse et al., 2012; https://www.arb-silva.de/aligner/) and a phylogenetic tree built with FastTree (Price et al., 2010). Trees were visualized with the Interactive Tree of Life (Letunic and Bork, 2007) and GGTREE (Yu et al., 2017). For calculating OTU and sequence percentages shared between trenches, samples were, in order, (1) rarefied to even sequence depth across samples, (2) pooled by trench of collection, and (3) the pooled groups rarefied again to account for different numbers of samples and sequencing depths between trenches. Because of the possibility of losing rare OTUs through pruning and rarefaction (McMurdie and Holmes, 2014), an analysis was also performed where low-abundance OTUs were not removed as described above (at least three reads in at least four samples) but instead only had to be in abundances greater than three reads across the entire dataset. Shared OTU and sequence percentages were then calculated by pooling samples within each trench and rarefying the combined trench communities to even total sequencing depth. This analysis therefore maintained rare OTUs and did not discard large numbers of sequences through rarefaction of each individual sample, but ignored the influence of certain samples having higher sequencing depths than others. For calculating shared OTU percentages between cores, samples were, in order, (1) rarefied to even sampling depth, (2) combined within 0–5 and 5–10 cm groupings within the same core of collection, (3) rarefied to even sampling depth again, and (4) combined by core. This was done to account for different sediment horizon depths, number of samples, and sequencing depth from influencing the abundances of OTUs, although at the cost of losing sequencing depth through rarefaction.

### Isolation and Characterization of Bacteria

*Bacteria* were cultured from sediment following plating onto agar or inoculation into pressurizable bulbs (Yayanos, 2001) and incubated at 4°C. Sediment samples from the top 1 cm of sediment, or a homogenized sample from the Rock Grabber, were diluted into 0.1-μm filtered trench water. Samples from the Kermadec Trench were incubated in 2216 Zobell Marine Medium (BD Difco™), A1 Medium (5 g potato starch, 2 g yeast extract, 1 g peptone, 0.5 g NH_4_Cl, 0.01 g L-methionine, 500 ml 0.2 μm-filtered autoclaved seawater collected from the Ellen Browning Scripps Pier), or a seawater minimal medium (10 mM NH_4_Cl, 14 mg Na_2_HPO_4_, American Type Culture Collection (ATCC) vitamin supplement (ATCC, Manassas, VA), ATCC trace mineral supplement, 1 L 0.2 μm-filtered Scripps Pier seawater), while samples from the Mariana Trench were inoculated in 2216 Marine Medium only. For samples to be incubated at high pressure, the media described above was inoculated, mixed with gelatin (pH 7 in sterile seawater) to a final concentration of 4% (Yayanos, 2001), transferred into polyethylene transfer pipettes (Samco, Thermo Fisher Scientific), heat sealed, and placed inside pressure vessels and incubated at the desired pressure at 4°C. Kermadec Trench samples were incubated at 100 MPa while those from the Mariana Trench were incubated at *in situ* pressure (40–110 MPa). After approximately 2 months, colony-forming units (CFUs) were calculated and representative isolates selected for 16S rRNA identification. Colonies arising at high pressure in bulbs were picked using a sterile syringe needle, inoculated into liquid medium, and grown until turbid at the original pressure of incubation at 4°C. Selected colonies were boiled and lysate used as template for PCR using the primers 27F and 1492R (Lane, 1991). Partial sequenced reads of at least 500 bp were identified using the SILVA database and compared to known sequences within NCBI.

## Results

### Community Composition and Diversity

**Figure 1:**
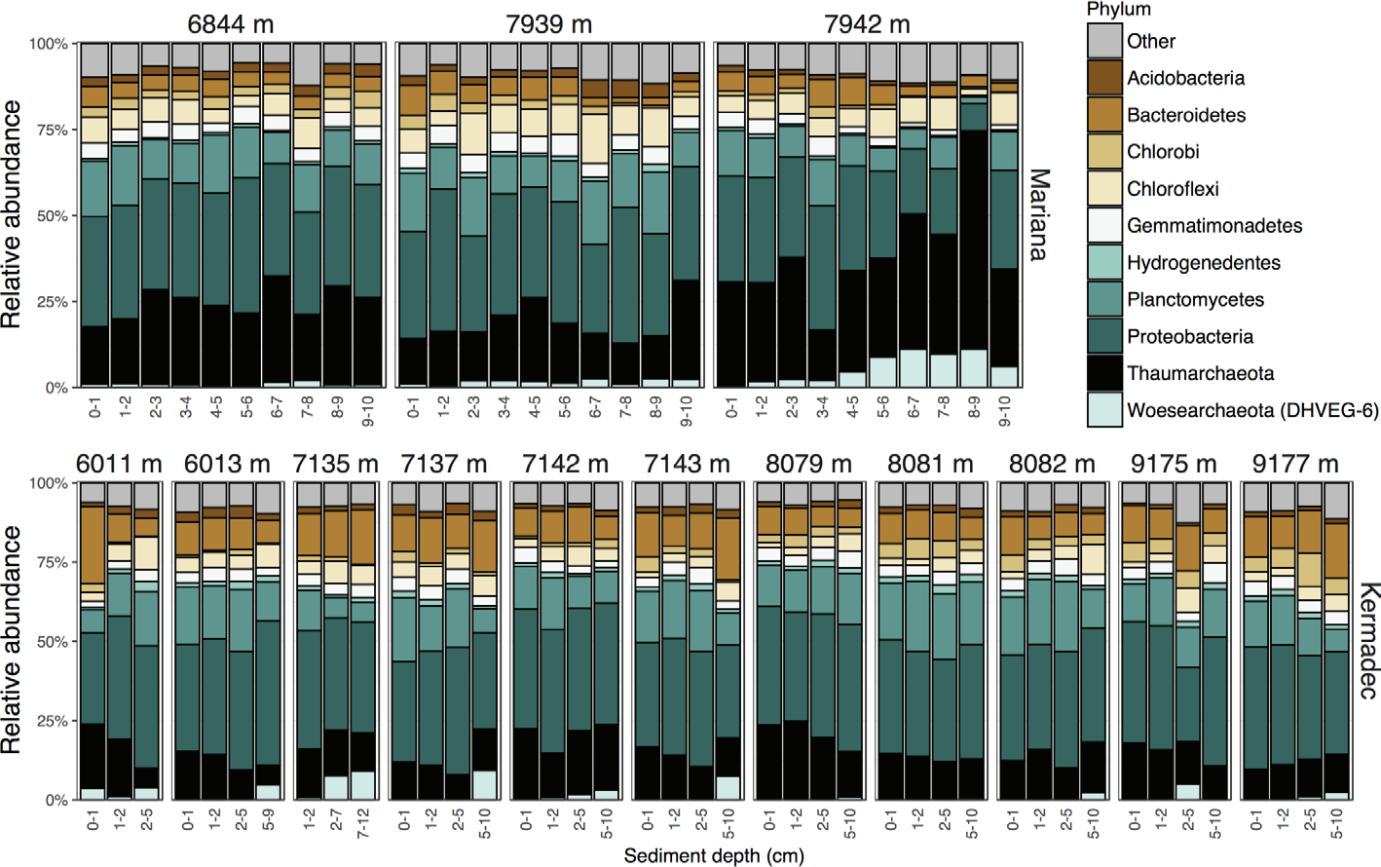
Relative sequence abundances of the 10 most abundant phyla within surficial sediments of the Kermadec and Mariana trenches organized by core (labeled by water depth), trench, and sediment horizon depth.

**Figure 2:**
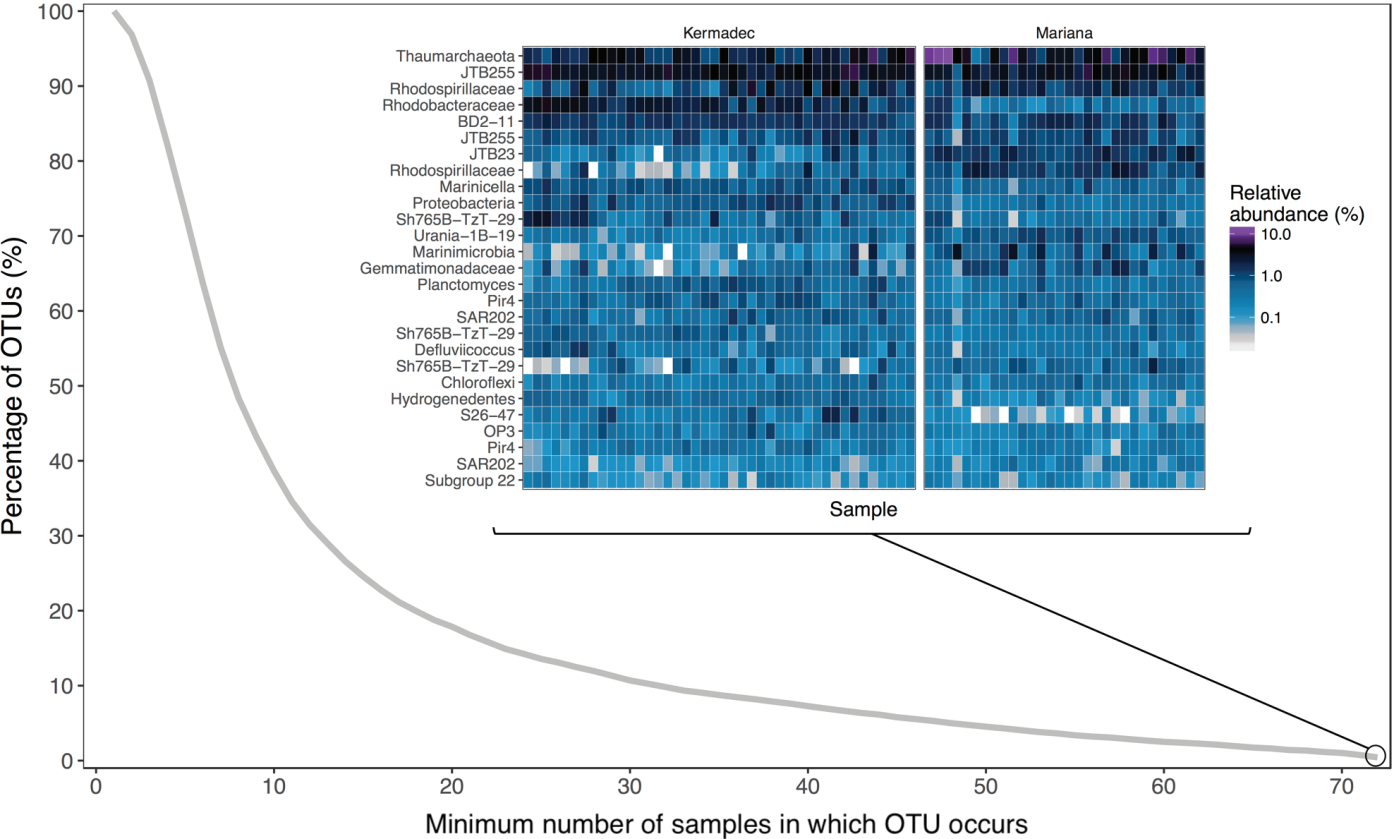
The percentage of the total operational taxonomic units (OTUs) as a function of the minimum number of samples in which they occur. The inset is a relative sequence abundance heat map of OTUs found in all 72 pristine core samples, organized in descending order of average relative sequence abundance across all samples. Labels represent the lowest identifiable taxonomic rank.

Hadal trenches are geographically isolated and can have different overlying primary productivity and water mass inputs that may lead to variations in community composition. Therefore, we evaluated the microbial communities within sediments from the Kermadec and Mariana trenches using high-throughput 16S rRNA gene sequencing of 88 samples. Seventy-two of these were from 14 pristine depth-fractioned cores, while 16 were from potentially disturbed sediments and were not included in the comparative analysis of the *in situ* communities here (Supplementary Figure 1, Supplementary Table 1). Rarefaction resulted in 7,231 sequences per sample and 6,317 total operational taxonomic units (OTUs) at 97% 16S rRNA V4-V5 gene sequence similarity. Phyla with high relative sequence abundances within the sediment samples included the *Proteobacteria*, *Thaumarchaeota*, *Bacteroidetes*, *Planctomycetes*, and *Chloroflexi* (Figure 1). While the majority of OTUs were seen in only a few samples each, 27 OTUs were present in all samples in both trenches (Figure 2). These shared OTUs included members related to the Marine Group I (MGI) within the *Thaumarchaeota*, the JTB255 clade and the genus *Marinicella* within the *Gammaproteobacteria*, the *Rhodobacterales* and *Rhodospirillales* within the *Alphaproteobacteria*, the class JTB23 within the Proteobacteria, and BD2–11 within the *Gemmatimonadetes* (Figure 2, Supplementary Table 2). On average, these OTUs made up 23 ± 4.5% (range 13.7–35.9%) of the total reads in each community and were closely related to sequences from previous studies of abyssal and hadal sediments (Supplementary Figure 2).

**Figure 3:**
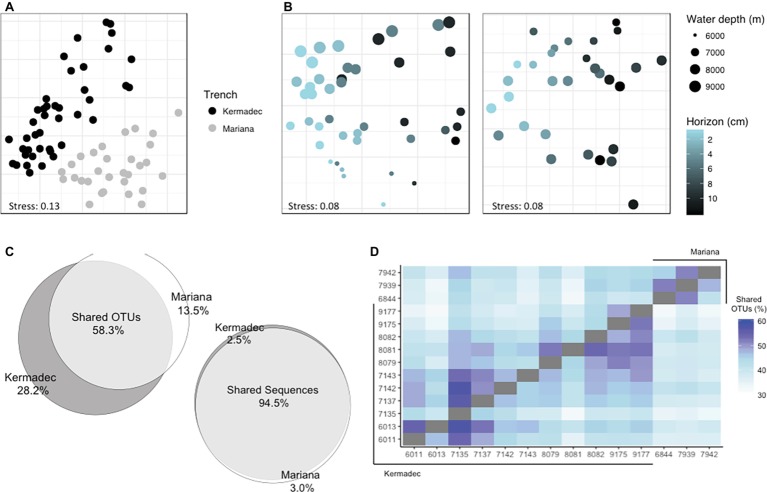
Distances between sediment communities visualized using NMDS ordinations of Bray-Curtis dissimilarities, labeled by trench **(A)** or separated by trench to examine the importance of sediment and water depth; **(B)** Left, Kermadec Trench; Right, Mariana Trench. **(C)** The percentage of OTUs and sequences shared between trenches. **(D)** The percentage of OTUs from one core found within another core, where the number of shared OTUs between samples on the x and y axes are shown as a percentage of the total OTUs found in the core on the x-axis.

Sediment community diversity was structured by trench of collection (Figures 3A,B, Supplementary Figure 4; PERMANOVA, *p* < 0.001, *R*^2^ = 0.23). Fifty-eight percent of OTUs, representing 95% of all sequences, were seen in both trenches (Figure 3C). When the only criterion for maintaining reads was that they had an abundance of at least three across the entire sediment dataset and samples were not rarefied, 40% of the OTUs were shared and still represented greater than 90% of all sequences. More OTUs from any given core could be found within cores from the same trench than cores from the other trench (Figure 3D; minimum found within a comparison core 32%, maximum 58%). Within both trenches assemblages varied by sediment horizon depth (Figure 3B; Kermadec, *p* < 0.001, *R*^2^ = 0.16; Mariana, *p* < 0.001, *R*^2^ = 0.21). Community richness was lower in deeper sediment horizons (Supplementary Figure 3). Community composition also varied by sampling site within each trench (Kermadec, *p* < 0.001, *R*^2^ = 0.25; Mariana, *p* < 0.001, *R*^2^ = 0.18) and water column depth (Kermadec, *p* < 0.001, *R*^2^ = 0.15; Mariana, *p* < 0.001, *R*^2^ = 0.15). Overall these parameters explained half of the variability within each trench.

### Taxa Distribution as a Function of Trench, Sediment Horizon Depth, and Water Depth

**Figure 4:**
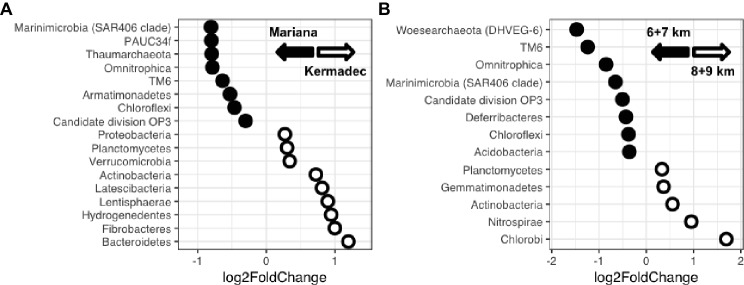
**(A)** Phyla showing enrichment within either the Kermadec or Mariana Trench. **(B)** Phyla showing enrichment at either upper hadal (6 + 7 km) or lower hadal (8 + 9 km) water depths in the Kermadec Trench.

Taxa that showed the largest differences in relative sequence abundances between trenches, sediment horizon depths, and water depths were identified. The Kermadec Trench was enriched in the phyla *Bacteroidetes*, *Hydrogenedentes*, *Planctomycetes*, and *Proteobacteria*, while the Mariana had higher abundances of the *Marinimicrobia*, *Thaumarchaeota*, *Woesearchaeota*, and *Chloroflexi* (Figure 4A, Supplementary Figure 5). The OTUs that varied the most between the Mariana and Kermadec trenches belonged to the MGI *Thaumarchaeota*, with specific OTUs showing enrichment in the Mariana Trench (Supplementary Figure 5). An OTU related to the genus *Aquibacter* reached abundances up to 11% of certain sediment horizons and was enriched in the Kermadec Trench. This *Aquibacter* OTU is identical to that which was found to be more abundant in the Kermadec Trench pelagic community than in the Mariana waters (Peoples et al., 2018a) and likely represents a new, hadal-adapted species (Peoples et al., 2018b).

**Figure 5:**
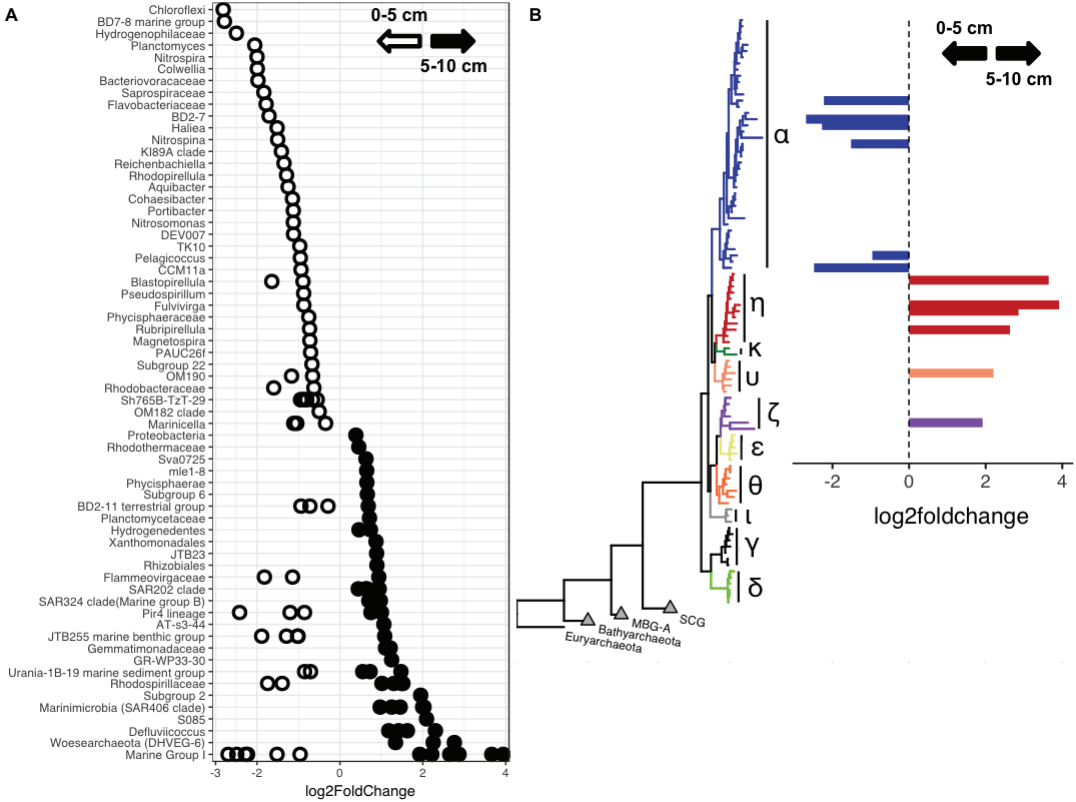
**(A)** OTUs showing enrichment within shallower (0–5 cm) or deeper (5–10 cm) sediments. The lowest identifiable taxonomic rank is shown. **(B)** *Thaumarchaeota* OTUs enriched at different sediment horizon depths, labeled by clade as described in Lauer et al. (2016).

Comparisons of surficial (0–5 cm) and deep (5–10 cm) sediments showed that taxa belonging to *Woesearchaeota*, *Marinimicrobia*, and *Chloroflexi*, including members of the clades S085, JG30-KF-CM66, SAR202, and *Anaerolineae*, were enriched in the deeper sediments (Figure 5A, Supplementary Figure 5, Supplementary Table 3). Phylogenetic analysis of *Marinimicrobia* OTUs enriched in the deeper sediment horizons showed they are related to sequences predominantly affiliated with deep-ocean sediments (Supplementary Figure 6). In contrast, the shallower sediments were enriched in classes belonging to the *Verrucomicrobia*, *Planctomycetes* and *Nitrospirae*, and OTUs belonging to the *Proteobacteria*, including *Colwellia*, and *Bacteroidetes*. MGI *Thaumarchaeota* showed strong variation by sediment horizon depth, with certain OTUs reaching up to 45% of some sediment horizon depths. Phylogenetic analysis indicated differentially abundant OTUs belonged to distinct clades, with members of the MGI alpha (α) subgroup more abundant in the upper sediments, while those belonging to the eta (η), upsilon (υ), and zeta (ζ) subgroups were enriched in deeper sediments (Figure 5B).

Sediment community composition also varied with water depth. Within the Kermadec Trench, higher percentages of OTUs within a given core could be identified within other cores from similar water depths (Figure 3D). When comparing cores as a function of water depth in the Kermadec Trench, the *Chlorobi*, *Nitrospirae*, and *Actinobacteria* were more abundant in the 8- and 9-km samples, while the *Chloroflexi*, *Acidobacteria*, and uncharacterized lineages were enriched at 6 and 7 km (Figure 4B). *Cyanobacteria*, which could be deposited through topographical funneling, were present, but not found at high abundances regardless of depth. However, an OTU related to the ML635J-21 group of *Cyanobacteria* was identified at relative sequence abundances of less than 0.1%. This OTU showed highest similarity to taxa from the Japan Trench (99%) and deep-ocean sediments from the Okinawa Trough (99%), with the next closest sequences being only 94% similar.

### Isolates and their *in situ* Abundances

**Figure 6:**
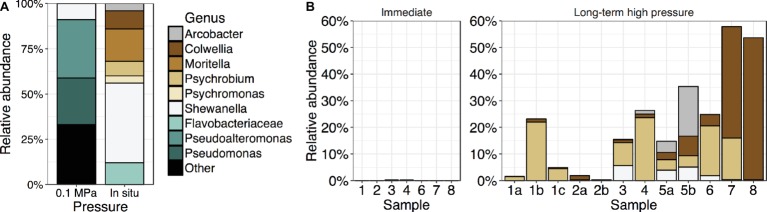
**(A)** Relative abundances of cultured isolates obtained with nutrient amendment at either 0.1 MPa on plates (*n* = 119) or high hydrostatic pressure in gelatin bulbs under *in situ* conditions (*n* = 50) at 4°C. **(B)** Relative sequence abundances within whole sediment communities of taxa related to high-pressure isolates (same colors as in A) in either *in situ*, immediately extracted sediment samples or the same samples after long-term high-pressure static incubations without nutrient amendment.

*Bacteria* were isolated under both low and high hydrostatic pressures to determine the diversity of culturable isolates. Fifty isolates obtained at high pressures under the facultatively anoxic conditions that develop in bulbs included members of the genera *Colwellia*, *Shewanella*, *Moritella*, and *Psychromonas* (Figure 6A, Supplementary Table 4). Isolates were also obtained from the genus *Psychrobium* within the *Gammaproteobacteria*, *Arcobacter* within the *Epsilonproteobacteria*, and a member of the *Flavobacteriaceae*, although none of these isolates survived cryopreservation or repeated subculturing. Qualitatively, the number of isolates obtained per sample was higher from the Kermadec than the Mariana Trench. This is consistent with the pristine sediment community sequence data where OTUs related to previously culturable piezophilic taxa represented ~0.20% of Kermadec samples but less than 0.05% in the Mariana Trench (Supplementary Figure 7; *t*-test, sediment only, *p* < 0.115; sediment and water from Peoples et al., 2018a, *p* < 0.001). One sample from the Kermadec Trench had relative sequence abundances of these taxa in excess of 4.8%. Relative sequence abundances of taxa related to culturable piezophiles were highest in the Kermadec Trench 0–1-cm fraction and lower in deeper sediments (*t*-test, Kermadec Trench, *p* < 0.003). No trend was seen among the altogether low relative sequence abundances present in the Mariana samples. Isolates obtained at atmospheric pressure on plates, rather than at high pressure, were related to the genera *Pseudoalteromonas*, *Pseudomonas*, *Halomonas*, and *Shewanella*. On average in both trenches OTUs related to these genera made up 0.18% relative sequence abundances within each sample.

### The Effects of Long-Term Pressurization

Sediment samples were maintained at 4°C under static, unamended, *in situ* pressures for longer than 1 year (hereafter called “long-term” samples) to test the effects of long-term sample incubation on community composition. These analyses were performed with the cores previously described and a number of other samples, including those collected using Van-Veen grabs, that may have been mixed with deeper sediment horizon depths and the overlying water (Supplementary Table 5). The resulting communities were compared to those from the same samples immediately frozen upon collection (“immediate” samples; Supplementary Table 5). DNA gram sediment^−1^ and alpha diversity were significantly lower in long-term samples relative to immediate samples (Supplementary Figure 8; *t*-test, DNA gram sediment^−1^, *p* < 0.035; Chao1, *p* < 0.004; Shannon, *p* < 0.001). OTUs related to the genera *Psychrobium*, *Colwellia*, *Arcobacter*, and *Shewanella* were enriched after long-term incubation, in some cases representing above 50% of the community despite starting abundances ranging from 0.0 to 0.2% (Figure 6). These OTUs were most similar to cultured piezophiles, including *Colwellia marinimaniae* (Kusube et al., 2016), *Colwellia piezophila*, and *Shewanella benthica* KT99, and to a member of the *Psychrobium* previously enriched after long-term pressurization (Aoki et al., 2014). Other long-term enriched taxa included members of the *Gammaproteobacteria*, *Rhodobacteraceae*, *Planctomycetes*, and *Bacteroidetes* (Supplementary Figure 9). In contrast, the long-term pressurized samples showed relative decreases in taxa belonging to uncharacterized and uncultivated lineages, including members of the clades BD2–11, BD7–8, NS72, SAR324, SAR202, *Marinimicrobia*, and *Omnitrophica* (OP3), and putative deep-ocean taxa such as *Defluviicoccus* and *Rhodospirillaceae*.

## Discussion

### Similar Lineages Are Present in both Mariana and Kermadec Trench Sediments

In this study, we evaluated the microbial community composition within trench sediments to test the hypothesis that hadal zones have distinct microbial communities from one another. While sediment communities were observed to be distinct between trench of collection and sampling site, there was significant overlap in the abundant taxa found in the Mariana and Kermadec trenches. Shared OTUs within both trenches represented greater than 90% of all reads. Therefore trench endemic OTUs made up small proportions of the communities when evaluated at 97% similarity. Many abundant taxa were closely related to members identified within other abyssal and hadal samples. These taxa belonged to lineages previously identified as having cosmopolitan members at bathyal and abyssal depths (Bienhold et al., 2016; Mußmann et al., 2017), such as sequences related to JTB255, BD2–11, JTB23, and the genus *Marinicella*. These results indicate that members within these lineages are present even at hadal depths. Similarly, the isolates obtained at high hydrostatic pressures were related to the genera *Colwellia*, *Shewanella*, and *Moritella*, consistent with previous studies. Many of the partial 16S rRNA genes of isolates from the present study were more than 97% similar to those of other piezophilic isolates. New strains were also obtained, including isolates related to the genus *Psychrobium* within the *Gammaproteobacteria*, *Arcobacter* within the *Epsilonproteobacteria*, and a member of the *Flavobacteriaceae.* Unfortunately, none of these isolates survived cryopreservation or repeated subculturing. Still, this *Psychrobium* isolate was related to that previously enriched after long-term pressurization (Aoki et al., 2014). Altogether, our results demonstrate the possible dispersal of OTUs between two widely separated trenches. Deep-ocean currents may lead to the dispersal and deposition of microorganisms in sediments (Müller et al., 2014), such as Antarctic Bottom Water flowing between the Kermadec and Mariana trenches. It is also possible some members do not require dispersal between trenches, but may originate within abyssal sediments, a possibility not yet evaluated. In this scenario, the microbes in question would possess high fitness in both abyssal and trench zones, potentially spreading between the two environments *via* bottom currents, or perhaps through earthquake-induced mass-wasting deposition down slope (Oguri et al., 2013). If taxa endemic to specific trenches exist in the hadal settings examined, they must exist at the strain rather than the species level, be rare, were lost during sampling, or are present in patchy or sample-specific distributions and were missed by our sampling. Comparisons with microbial communities in more distant trenches, such as the Puerto Rico or Atacama trenches, may show higher sequence abundances of endemic taxa.

### Inter- and Intra-Trench Variation May Be Due to Organic Matter

Differences in organic matter due to primary production in overlying surface waters and its deposition through topographical funneling may be one of the most important factors structuring communities within trenches (Danovaro et al., 2003; Glud et al., 2013; Ichino et al., 2015; Wenzhöfer et al., 2016). The Kermadec Trench may have higher concentrations of organic matter than the Mariana Trench because of differences in primary productivity at the surface (Longhurst et al., 1995; Jamieson, 2015). While we do not have organic matter concentrations to report here for each core, preliminary data from the 0–1-cm fraction at 8000 m in each trench suggest that percent total organic carbon is higher in the Kermadec Trench (~0.5%) than the Mariana Trench (~0.4%; Grammatopoulou et al., in prep). Therefore, it is reasonable to hypothesize that organic matter is in part responsible for the differences between the Mariana and Kermadec trench communities. Consistent with this, the Kermadec Trench was enriched, relative to the Mariana Trench, in the *Bacteroidetes*, *Actinobacteria*, and *Proteobacteria*, lineages that have been found to correlate with higher concentrations of organic matter (Bienhold et al., 2012; Learman et al., 2016). In contrast, the Mariana Trench had higher proportions of *Thaumarchaeota*, *Chloroflexi*, and other uncharacterized lineages. Although *Archaea* are ubiquitous in marine sediments, organic matter concentrations are thought to negatively correlate with abundances of ammonia-oxidizing *Archaea* (Luo et al., 2015; Learman et al., 2016), potentially due to differences in electron acceptor availability or *Archaea* outcompeting *Bacteria* under energy-starved conditions (Valentine, 2007; Hoehler and Jorgensen, 2013). The *Chloroflexi* may also be adapted to degrading recalcitrant organic matter (Landry et al., 2017). Furthermore, the number of piezophilic isolates and related sequences in the high-throughput community data were higher within the Kermadec than the Mariana Trench and were enriched in the upper layers of sediment. Their enrichment in the Kermadec Trench pelagic community and on larger size fractions has been suggested to be a result of adaptations to higher concentrations of organic matter, including particulate forms (Peoples et al., 2018a). Intra-trench variability may also be influenced by organic matter due to topographic funneling into the axis of the trench. Within the Kermadec Trench the *Chloroflexi* and *Acidobacteria* were enriched at 6- and 7-km depths whereas the *Nitrospirae* and *Actinobacteria* are more abundant at 8- and 9-km depths. Enrichments of *Nitrospirae* within the hadal water column have been attributed to a more eutrophic environment (Nunoura et al., 2015). Altogether, these findings suggest the Kermadec Trench may be enriched in organic matter relative to the Mariana Trench. Ultimately, deep-sea microbial communities are governed by a myriad of variables that may contribute to these inter- and intra-trench differences, such as hydrography (Hamdan et al., 2013), specific location of sample collection, temporal variability and season of sample collection (Lampitt, 1985), sediment lithology (Probandt et al., 2017), or the quality, not just the quantity, of organic matter present. Our findings support the notion that organic matter can contribute to the spatio-temporal variability in deep-sea microbial communities.

### Centimeter-Scale Sediment Depth Changes Select for Certain Microbial Lineages

While trench sediments may become mixed and suspended due to tectonic activity and topographic instability (Kawagucci et al., 2012; Oguri et al., 2013; Nunoura et al., 2016), the sediment communities studied here were stratified and compositionally distinct from those in the water column above them (Peoples et al., 2018a). Because the oxygen penetration depth can be limited to the top 10 cm in trenches (Glud et al., 2013; Wenzhöfer et al., 2016), we compared the microbial communities present in the shallower and deeper sediment horizons. Community richness and diversity varied with sediment horizon depth, consistent with previous comparisons of surficial and deep subsurface environments (Durbin and Teske, 2010; Teske et al., 2011; Shulse et al., 2016; Walsh et al., 2016a,b). Our findings suggest that specific deep-sea lineages are enriched within deeper marine sediments. Within the *Thaumarchaeota,* MGI-α showed enrichment in the upper sediment while those belonging to the MGI-η subgroup were more abundant in the deeper sediments. Such ecotype differentiation has been previously noted in abyssal sediments (Durbin and Teske, 2010; Tully and Heidelberg, 2013; Lauer et al., 2016) and may be due to changes in organic matter abundance or oxygen availability (Durbin and Teske, 2010). Abundances of alternative electron acceptors, such as nitrate, sulfate, or iron, influence community composition (D’Hondt et al., 2009; Durbin and Teske, 2010, 2012; Nunoura et al., 2013; Jessen et al., 2017) and therefore likely play an important role in hadal sediments (Nunoura et al., 2013, 2018) as the oxygen penetration depth can be shallow (Glud et al., 2013; Wenzhöfer et al., 2016). Relative sequence abundances of the *Woesearchaeota* increased with increasing sediment horizon depth. This archaeal phylum has members that are specifically enriched in anoxic niches and can have fermentative or symbiotic lifestyles (Castelle et al., 2015; Lazar et al., 2017; Liu et al., 2018). Differences in genomic potential and niche separation of *Woesearchaeota* suggest that unexplored diversity exists within this phylum (Shcherbakova et al., 2016; Narrowe et al., 2017). OTUs related to the *Woesearchaeota* within our dataset were remarkably distinct from other previously identified studies, showing highest similarity to sequences from abyssal and hadal sites and low (<95%) similarity to representative sequences from other habitats (Supplementary Figure 10). The *Marinimicrobia*, which are abundant within the deep-ocean water column (Nunoura et al., 2015; Tarn et al., 2016; Peoples et al., 2018a), also increased with sediment horizon depth. Based on phylogenetic analysis (Supplementary Figure 6) the *Marinimicrobia* OTUs identified here belong to deep-sea sediment-associated clades that have not been previously identified (Hawley et al., 2017).

### Current High-Pressure Culturing Methods Are Inappropriate for Most Deep-Sea Taxa

Although a large diversity of *Bacteria* and *Archaea* were found within the sediments, very few taxa were successfully isolated. Many of those that were belong to the same genera as known piezophiles, consistent with most previous isolation attempts from the deep sea. Interestingly, after unamended, long-term static pressurization of sediment samples, higher relative sequence abundances of taxa related to the cultured piezophiles *Psychrobium*, *Colwellia*, *Arcobacter*, and *Shewanella* were found. In shallow-ocean communities, *Colwellia* is one of the first responders to microcosm conditions (Stewart et al., 2012; Mayali et al., 2016) and enrichments of *Colwelliaceae*, *Moritellaceae*, *Psychromonadaceae*, and *Shewanellaceae* have been observed within enrichments of Arctic bathyal-depth sediment samples under high pressure (Hoffmann et al., 2017). Therefore, regardless of location, depth of collection (surface, bathyal, and in this study hadal), nutrient enrichment, or temperature or pressure incubation conditions, taxa belonging to the same heterotrophic and copiotrophic genera within the *Gammaproteobacteria* are enriched under mesocosm conditions. These taxa may contain metabolic versatility for colonizing various ecological niches during fluctuating environmental conditions (Stewart et al., 2012). In contrast, microbial populations from immediately recovered samples were enriched in taxa belonging to uncharacterized and uncultivated lineages representative of the *in situ* diversity. Taken together with the enrichment of culturable taxa, these findings highlight the difficulties in obtaining pure cultures of representative and novel deep-ocean lineages. This study is the first to show that long-term incubations in pressure vessels, regardless of nutrient amendment or collection location, clearly select for the same piezophilic taxa. Decompression during sample retrieval, static incubation conditions in pressure vessels leading to lack of oxygen or other important nutrients, or eukaryotic predation (Tsagaraki et al., 2018) may significantly bias our ability to obtain cultures of piezophiles.

## Conclusions

Here, we present the first comparison of microbial communities within hadal sediments of two trenches. Sediment community composition varied by trench of collection, sediment horizon depth, and water column depth, changes predicted to be due in part to variations in organic matter concentrations. Future studies of hadal sediments should couple their analyses of community composition to organic matter concentrations and compositions. While the communities differed between the two trenches, neither appeared to be dominated by endemic microbial communities at the 97% OTU level. Instead, the hadal sediments shared many cosmopolitan taxa similar to those found in other abyssal and hadal sites, suggesting members of these lineages may be ubiquitous at hadal depths. These findings highlight the possibility of microbial dispersal over long distances between hadal zones. Whether these taxa are actually distinct, both between trenches and from those found at abyssal sites, will ultimately require whole genome comparisons and analyses of phenotypic plasticity, and not just partial 16S rRNA gene analyses. Culturing and *in situ* abundances of known piezophiles showed that these taxa represent a relatively small fraction of environmental samples and were enriched in the Kermadec Trench, perhaps because of affiliation with organic-rich conditions. After long-term batch incubation of sediments at *in situ* high hydrostatic pressure, these taxa came to dominate the communities at the expense of initially more abundant members. The attempted isolation of piezophiles extends back to the 1940s and yet very few taxa have been isolated, potentially because of the use of pressure vessels. Future work should attempt to more closely mimic *in situ* conditions using recirculating systems or, perhaps more effectively, attempt to enrich for microorganisms *in situ*, as current practices involving removing samples from the deep-ocean ultimately select for a few taxa that are not representative of deep-ocean sediment communities at large.

## Author Contributions

LP and DB were involved in experimental design. LP, EG, CN, JD, DM, and DB collected the samples. LP, EG, MP, XX, and OO performed the research. JB and EA provided methodological and technical advice. LP and DB wrote the manuscript. All authors approved the final version.

### Conflict of Interest Statement

The authors declare that the research was conducted in the absence of any commercial or financial relationships that could be construed as a potential conflict of interest.
