# Supplementary material for: Microbial Community Diversity Within Sediments from Two Geographically Separated Hadal Trenches
Source: Front Microbiol. 2019 Mar 15;10:347. doi: 10.3389/fmicb.2019.00347 (PMC6428765; doi:10.3389/fmicb.2019.00347)
Supplement: Supplementary file 1 [file Data_Sheet_1.docx]

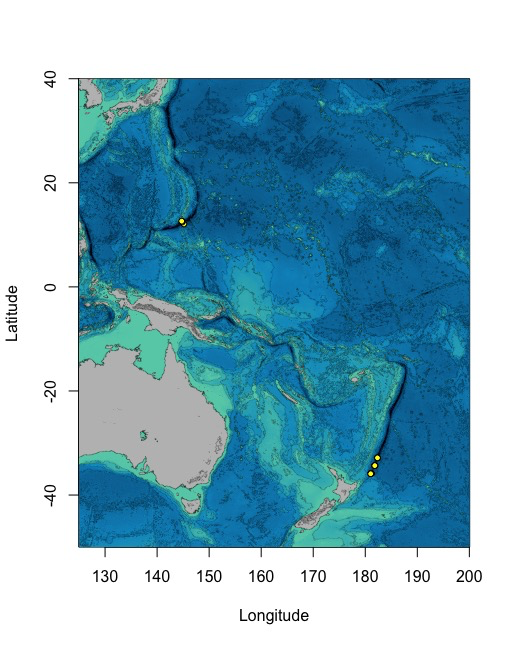


Supplementary Fig. 1. Locations within the Mariana and Kermadec trenches where pristine core samples were collected.

Supplementary Fig. 2. Ribosomal 16S RNA gene phylogenetic tree of representative abundant OTUs and closely related taxa.

Supplementary Fig. 3. A+B; Concentrations of DNA extracted from either the Mariana (A) or Kermadec (B) Trench. C+D; Alpha diversity as a function of sediment depth in both trenches (Pearson correlation: Both trenches, Chao1, r=-0.71, p<.0001; Both trenches, Shannon, r=-0.61, p<.0001).

Supplementary Fig. 4. NMDS ordinations (stress: 0.13) of pristine core samples colored by core (top; ‘Description’, where ‘CR’ cores are from the Mariana and ‘N0’ cores are from the Kermadec), water column depth (middle), and sediment horizon depth (bottom).


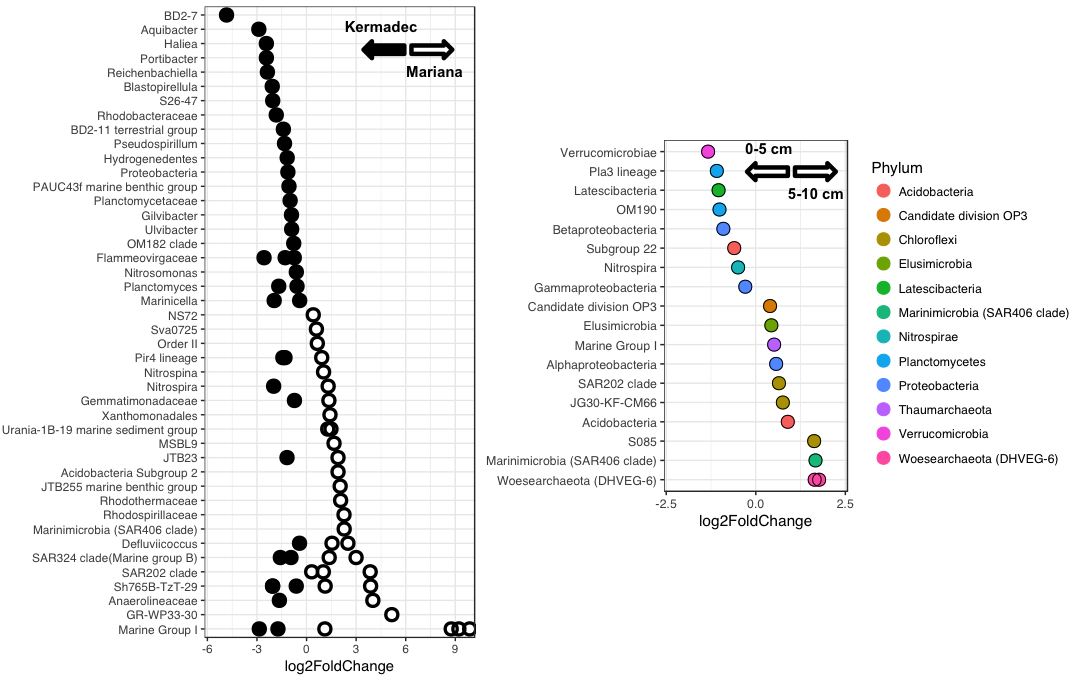


Supplementary Fig. 5. Left; OTUs enriched within either the Mariana or Kermadec trenches. Right; Classes enriched within either the upper (0-5 cm) or lower (5-10 cm) sediment horizon depths. The lowest discernible taxonomic ranks are shown in all cases.

Supplementary Fig. 6. Phylogenetic tree of the *Marinimicrobia* phylum, with clades containing predominantly sediment-associated sequences in brown. Red sequences are those obtained in this study.

Supplementary Fig. 7. Relative abundances of piezophilic *Colwellia, Shewanella, Moritella,* and *Psychromonas* within the pristine core samples using high-throughput 16S rRNA gene sequencing. Water samples include abundances from data described in Peoples *et al*., 2018a. One outlying point from the Kermadec Trench at 2–5 cm depth had an abundance of 4.8% (not shown) but was excluded from statistical analysis as it varied by over two standard deviations from the mean. Significant differences were identified between the Kermadec >3.0 and 3.0–0.2 um fractions (t-Test: p<0.046), Kermadec >3.0 and Mariana >3.0 um fractions (p<0.041), Kermadec 0–1 and 1–10 cm fractions (p<0.003), and all Kermadec and all Mariana samples (p<0.001).

Supplementary Fig. 8. A; Concentrations of DNA extracted from sediments immediately after collection or those same sediments after long-term pressurization. Not all samples shown were successfully sequenced or analyzed for their community composition. B; Alpha diversity metrics comparing samples extracted immediately or after long-term pressurization.

Supplementary Fig. 9. Differentially abundant OTUs when comparing samples that had been extracted immediately or those same samples after long-term pressurization. Negative log2fold change, immediate *in situ*; positive, long-term pressurization. The lowest identifiable taxonomic rank is shown.

Supplementary Fig. 10. Phylogenetic tree of the *Woesearchaeota*, with sequences labeled based on subgroups determined by Liu *et al*., 2018. Sequences in bold were those obtained in this study.
